# Data on glycerol/tartaric acid-based copolymer containing ciprofloxacin for wound healing applications

**DOI:** 10.1016/j.dib.2016.04.010

**Published:** 2016-04-11

**Authors:** E. De Giglio, M.A. Bonifacio, S. Cometa, D. Vona, M. Mattioli-Belmonte, M. Dicarlo, E. Ceci, V. Fino, S.R. Cicco, G.M. Farinola

**Affiliations:** a Department of Chemistry, University of Bari Aldo Moro, Via E. Orabona 4, 70126 Bari, Italy; b Jaber Innovation srl, via Calcutta 8, 00100 Rome, Italy; c Department of Clinical and Molecular Sciences, Università Politecnica delle Marche, Via Tronto 10/a, 60020 Ancona, Italy; d Department of Veterinary Medicine, University of Bari Aldo Moro, Str. Prov. per Casamassima Km 3,Valenzano, BA, Italy; e Synchimia srl, Spin-off of University of Bari Aldo-Moro, Via Orabona, 4, 70126 Bari, Italy; f CNR-ICCOM Bari, Via Orabona, 4, 70126 Bari, Italy

**Keywords:** Glycerol-based copolymer, Differential Scanning Calorimetry (DSC), Fourier Transform Infrared Spectroscopy (FT-IR), Drug release

## Abstract

This data article is related to our recently published research paper “Exploiting a new glycerol-based copolymer as a route to wound healing: synthesis, characterization and biocompatibility assessment", De Giglio et al. (Colloids and Surfaces B: Biointerfaces 136 (2015) 600–611) [1]. The latter described a new copolymer derived from glycerol and tartaric acid (PGT). Herein, an investigation about the PGT-ciprofloxacin (CIP) interactions by means of Fourier Transform Infrared Spectroscopy (FT-IR) acquired in Attenuated Total Reflectance (ATR) mode and Differential Scanning Calorimetry (DSC) was reported. Moreover, CIP release experiments on CIP-PGT patches were performed by High Performance Liquid Chromatography (HPLC) at different pH values.

**Specifications table**

**Table:** 

Subject area	*Material science. Chemistry.*
More specific subject area	*Biomaterials and drug delivery systems*
Type of data	*Data, table and figures description*
How data was acquired	*FT-IR in ATR mode were performed by Perkin-Elmer Spectrum Two (Perkin-Elmer Inc, Waltham, MA). ATR correction algorithm (included into the Spectrum software) was employed on all the presented spectra;*
*DSC measurements were performed by Perkin-Elmer DSC400 Calorimeter (Perkin-Elmer Inc, Waltham, MA);*
*HPLC analyses were performed with an Agilent 1260 Infinity Chromatograph (Agilent Technologies, Santa Clara, CA).*
Data format	*Analyzed*
Experimental factors	*No pre-treatment of samples was required*
Experimental features	*For ATR analysis, no specific sample preparation was required.*
*For DSC analysis, samples were heated at a constant rate of 10 °C/min, in a temperature range of 30-400° C.*
*For HPLC measurements, a drug-copolymer mixture (3:5 w/w ratio) was casted on nonwoven square tapes until dried. The prepared samples were immersed in physiological solution at two different pHs.*
Data source location	*Department of Chemistry, University of Bari Aldo Moro, Bari (Italy)*
Data accessibility	*Data is available with this article.*

**Value of the data**

• FT-IR spectra may help other researchers in the analysis of comparable polymeric structures.
• DSC data are useful to explain the CIP-PGT interactions.
• Data about CIP release from PGT-based patches provide insight into the antibiotic release at different pH values.

## 1 Data

FT-IR spectra acquired in ATR mode and DSC analysis are related to PGT, CIP and their mixture, while, HPLC measurements refer to CIP-loaded PGT casted on non-woven patches.

## 2 Experimental design, materials and methods

### 2.1 Materials

Chemical compounds used in this article: a new copolymer (PGT) was synthesized by using the following compounds: glycerol (PubChem CID: 753) and L-tartaric acid (PubChem CID: 444305), according to a procedure reported in [1]. Patches based on a non-woven tape impregnated by a mixture of PGT and ciprofloxacin hydrochloride (CIP) (PubChem CID: 62999) are proposed to provide a topical delivery vehicle to treat skin infections.

### 2.2 Fourier Transform Infrared Spectroscopy (FT-IR)

**Fig. 2:**
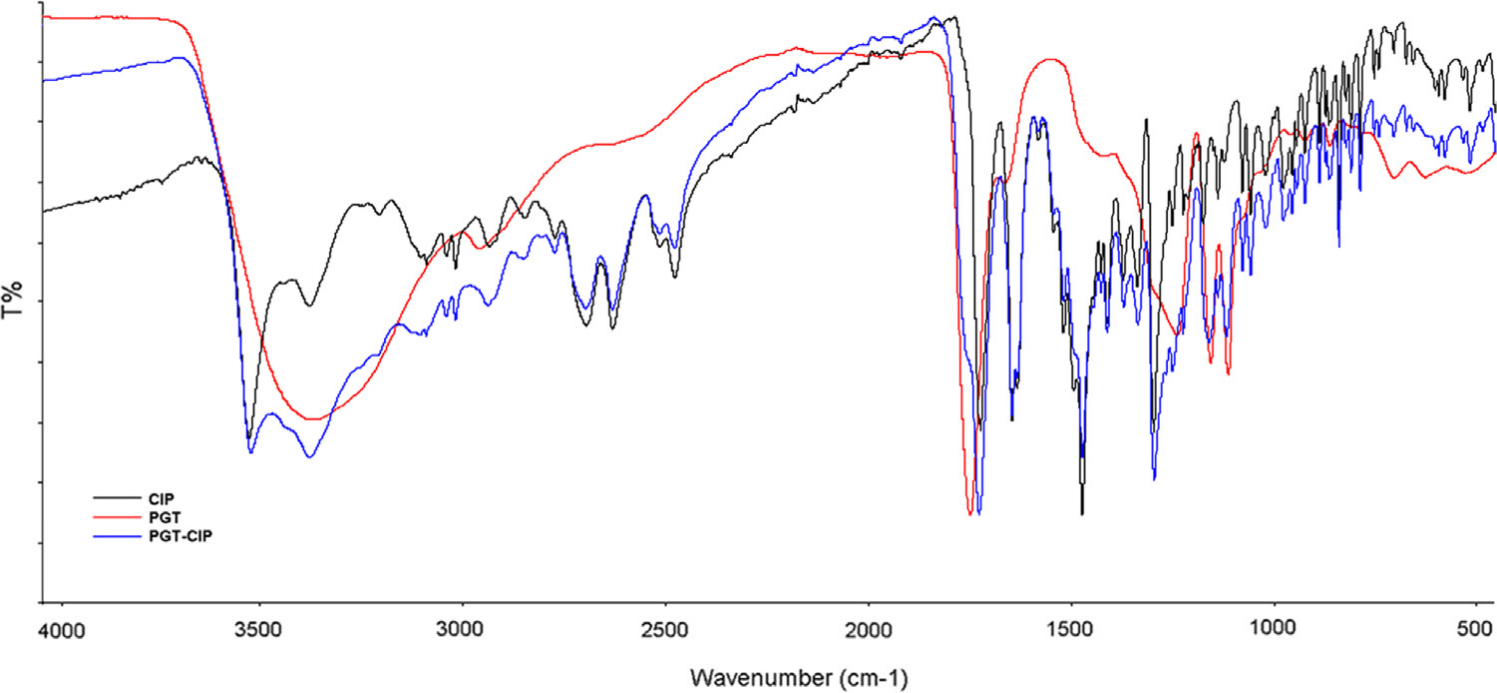
FT-IR analysis**:** ATR spectra of ciprofloxacin (CIP; black line), copolymer (PGT; red line) and their mixture (CIP-loaded PGT copolymer; blue line).

ATR spectra (Fig. 2) of CIP, PGT and CIP-loaded PGT (PGT/CIP 5:3 w/w ratio) were measured on a spectrum two Perkin-Elmer using the Universal ATR accessory (Single Reflection Diamond). The samples were analyzed without any preliminary preparative step. The peak wavenumbers and attributions of CIP, reported in Table 1, were in agreement with the literature data [2]. PGT copolymer and CIP-loaded PGT ATR peaks were also reported.

### 2.3 Thermal analysis by Differential Scanning Calorimetry (DSC)

DSC measurements were performed by a Perkin-Elmer DSC400, equipped with Pyris software for thermogram processing. Samples of 8–10 mg were placed in a flat bottomed aluminum pan and heated at a constant rate of 10 °C/min, using dry nitrogen atmosphere as carrier gas, in a temperature range of 30–400 °C. The instrument temperature and energy scales were calibrated using purified Indium (99.9%) as the standard reference material.

**Fig. 3:**
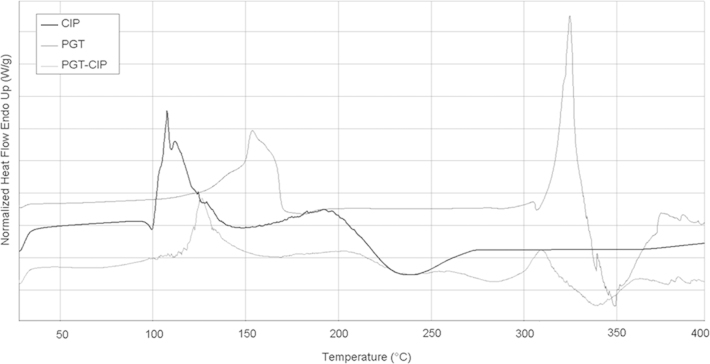
DSC analysis: thermograms of ciprofloxacin (CIP), copolymer (PGT) and their mixtures in the range 40–400 °C under nitrogen atmosphere.

The thermal analysis is a valuable method to investigate drug–polymer interactions and to optimize formulations of pharmaceutical dosage forms [3]. DSC of plain drug, PGT copolymer and CIP-loaded copolymer (PGT/CIP 5:3 w/w ratio), shown in Fig. 3, were performed to determine any change in the drug melting temperature, in order to investigate possible interactions between the drug and the copolymer.

The DSC data demonstrated endothermic peaks for CIP and PGT at 154 °C and 108 °C, respectively. The PGT–CIP mixture thermogram showed the disappearance of endothermic peaks of CIP and PGT and the appearance of another endothermic peak at 127 °C. CIP melting point was found at 325 °C followed by decomposition; this peak was anticipated to 310 °C when CIP was included in PGT gel.

### 2.4 Ciprofloxacin release from PGT-based patches at different pH values

CIP release was monitored dipping the PGT impregnated patches in 10 ml of physiologic solution (NaCl 0.9%) at pH 5.0 or pH 7.4, equilibrated to 37±0.5 °C in a thermostatic shaking incubator.

At pre-determined time intervals aliquots of 500 µl were withdrawn and replaced with fresh solution. CIP HPLC analyses were performed on an Agilent 1260 Infinity Chromatograph with a Multiple Wavelength (MW) detector, 20 µl injection loop. A reversed phase synergy column (15 cmx4.6 mm; 4 µm particles; Phenomenex) was eluted in isocratic mode, monitoring continuously the effluent at 280 nm [4]. A liter of mobile phase consisted of a mixture of 900 ml of 50 ml/L acetic acid, plus 50 ml acetonitrile and 50 ml methanol. The flow rate was maintained to 1.25 ml/min. Acquired data were processed by the Agilent Technologies ChemStation software for LC systems and compound quantification was carried out as previously reported [1].

**Fig. 4:**
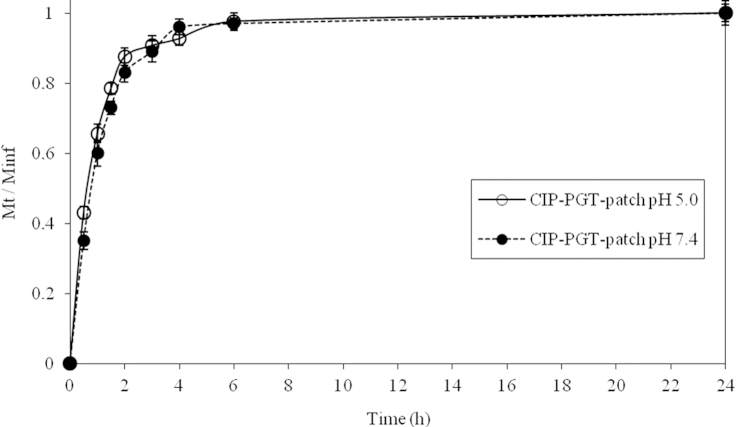
Drug delivery from PGT patches**:** ciprofloxacin cumulative release, expressed as Mt/M∞, from drug loaded PGT patches at two different pH values.

Solubility is one of the properties which directly impact on bioavailability of an active pharmaceutical ingredient. The aqueous solubility of CIP is pH-dependent [5]. At neutral pH, the zwitterionic and unionised forms of CIP are dominant. In this form, CIP presents the minimum solubility. [6] Pathogenic bacteria prefer the neutral environment (pH 7.4) that characterizes subcutaneous tissue to the slightly acidic one of integer skin surface (pH 5.0) [6]. In this respect, CIP release from drug loaded PGT patches was performed in NaCl 0.9%, 37 °C), varying only the medium pH. As shown in Fig. 4, no significant differences in the release profile were detected varying the pH of the release medium.

## Figures and Tables

**Fig. 1:**
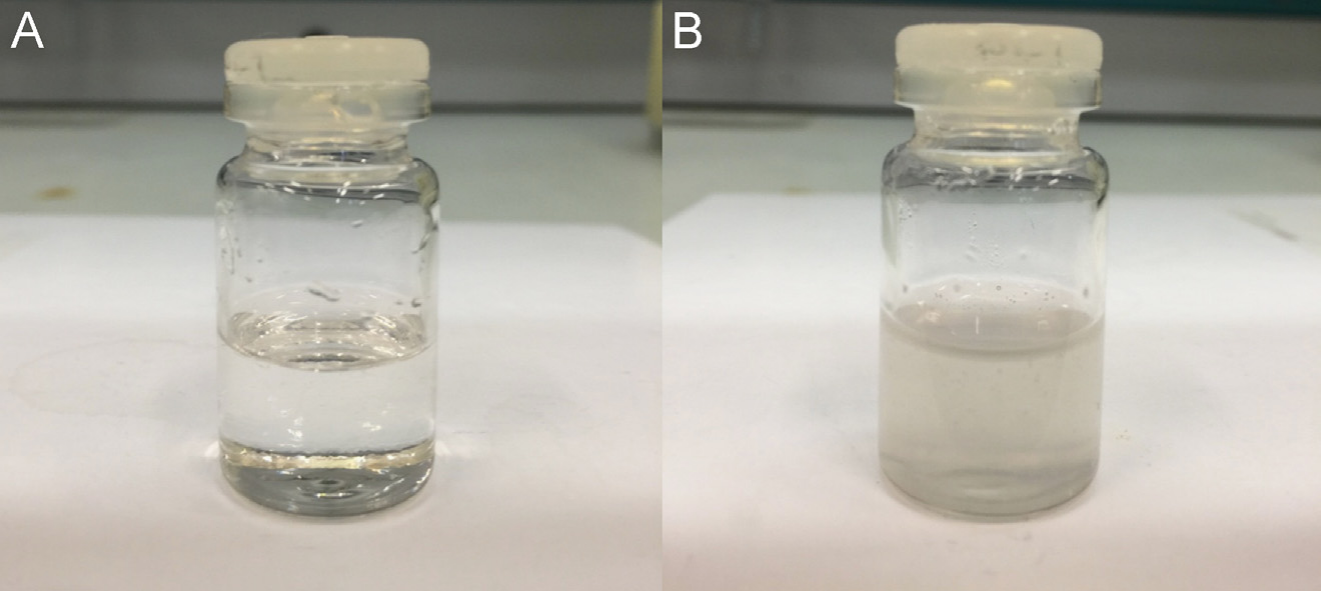
Photographs of PGT and CIP–PGT mixture**:** copolymer (PGT) and a mixture of PGT and ciprofloxacin (CIP) are shown in Fig. 1 A and B, respectively.

**Table 1:** ATR peak wavenumbers and assignments of CIP, PGT and PGT-CIP formulations.

**Prominent ATR peaks of CIP**	
**Peaks (cm**^**−1**^**)**	**Peaks assignment**
3500–3450	O–H stretching vibration
3000–2950	υ=CH and Ar–H
1699	C=O stretching vibration
1621	δN–H bending vibration
1446	υC–O
1307–1295	δO–H bending vibration
1050–1000	C–F stretching
**Prominent ATR peaks of PGT**	
**Peaks (cm**^**−1**^**)**	**Peaks assignment**
3350	O–H stretching vibration
2960–2930	–CH symmetric and asymmetric stretching
1727	C=O stretching vibration
1410	–C–H deformation vibration from asymmetric stretching
1270–1080	double vibrational stretching of –C–O and –O–C
1124	C–O stretching
**Prominent ATR peaks of CIP–PGT Formulation**	
**Peaks (cm**^**−1**^**)**	**Peaks assignment**
3350	O–H stretching vibration
3000–2950	υ=CH and Ar–H
2960–2930	–CH symmetric and asymmetric stretching
1727	C=O stretching vibration
1703	C=O stretching vibration
1621	δN–H bending vibration
1446	υC–O
1410	–C–H deformation vibration from asymmetric stretching
1307–1295	δO–H bending vibration
1270–1080	double vibrational stretching of –C–O and –O–C
1124	C–O stretching
1050–1000	C–F stretching
