# Supplementary material for: Data on glycerol/tartaric acid-based copolymer containing ciprofloxacin for wound healing applications
Source: Data Brief. 2016 Apr 11;7:1335–40. doi: 10.1016/j.dib.2016.04.010 (PMC4842849; doi:10.1016/j.dib.2016.04.010)
Supplement: Supplementary file 1 — Supplementary material [file mmc1.docx]

**Conflict of interest declaration**

The corresponding author on behalf of all other authors confirms that: (i) there are no known conflicts of interest associated with this publication and there has been no significant financial support for this work that could have influenced its outcome; (ii) the manuscript has been read and approved by all authors and that there are no other persons who satisfied the criteria for authorship but are not listed; (iii) the order of authors listed in the manuscript has been approved by everyone; (iv) due consideration has been given to the protection of intellectual property associated with this work and therefore there are no impediments to publication, including the timing of publication, with respect to intellectual property. In so doing I confirm that we have followed the regulations of our institutions concerning intellectual property.

All authors understand that the Corresponding Author is the sole contact for the Editorial process (including Editorial Manager and direct communications with the office). The corresponding author is responsible for communicating with the other authors about progress, submissions of revisions and final approval of proofs.


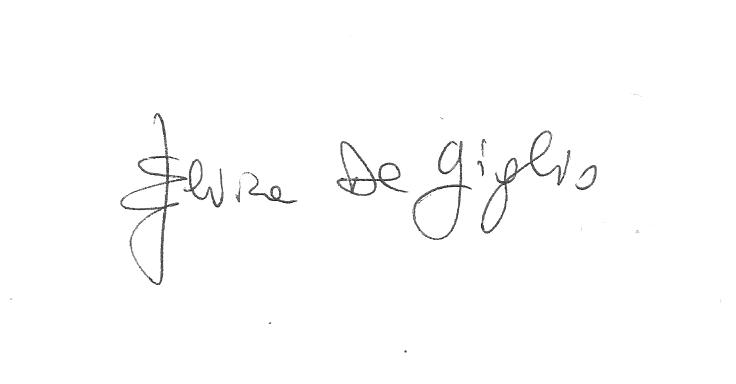
 The corresponding author
